# Performance of Ultra-High-Frequency Ultrasound in the Evaluation of Skin Involvement in Systemic Sclerosis: A Cross-Sectional Pilot Study

**DOI:** 10.3390/diagnostics15131600

**Published:** 2025-06-24

**Authors:** Olga Barbara Krammer, Martin Fleck, Boris Ehrenstein, Wolfgang Hartung, Florian Günther

**Affiliations:** 1 Department of Rheumatology and Clinical Immunology, Asklepios Hospital Bad Abbach, 93077 Bad Abbach, Germanyb.ehrenstein@asklepios.com (B.E.); w.hartung@asklepios.com (W.H.); f.guenther@asklepios.com (F.G.); 2 Department of Internal Medicine I, University Medical Center, 93053 Regensburg, Germany

**Keywords:** ultra-high-frequency ultrasound, skin assessment, systemic sclerosis

## Abstract

**Objective**: The aim of this study was to assess the performance and feasibility of ultra-high-frequency ultrasound (UHF-US) in clinical practice for measuring skin thickness in patients with systemic sclerosis (SSc) compared to age- and sex-matched controls. **Materials and Methods**: A total of 14 patients with SSc and 14 healthy controls (HCs) were enrolled in the study. All subjects underwent US evaluation of the epidermis, dermis and cutis by three experts in the 17 sites of the modified Rodnan skin score (mRSS). All the sonographers were blinded to the mRSS, which was assessed by an experienced rheumatologist who was not involved in, and blinded to, the US assessment. **Results**: In comparison to HCs, dermal thickness was significantly higher in patients at six sites: the right (*p* < 0.001) and left (*p* = 0.001) finger; right (*p* = 0.027) and left (*p* = 0.048) hand; left foot (*p* = 0.010) and face (*p* < 0.001). The epidermal layer did not differ significantly. At all mRSS sites except for the chest, there were moderate to strong positive correlations between US-assessed dermal thickness and local mRSS. The interobserver reliability for all sites of the mRSS, with the exception of the face, was good to excellent (with an intraclass correlation coefficient [ICC] ranging from 0.724 to 0.939). **Conclusions**: These data support the use of UHF-US as an objective and reliable tool for the assessment of skin involvement in patients with SSc. Considering its feasibility in clinical practice, we suggest that US assessment of skin in patients with SSc should be restricted to the dermal layer of the fingers and hands, since they are the sites that skin fibrosis typically starts from.

## 1. Introduction

Systemic sclerosis (SSc) is an autoimmune disease with a complex pathogenesis leading to diffuse microangiopathy and fibrosis of the skin and internal organs [1,2]. Early diagnosis and treatment, as well as close monitoring of patients, are essential for understanding the further course of the disease [3]. Skin involvement is characterized by thickening and hardening of the skin due to increased dermal collagen deposition. Skin fibrosis not only affects quality of life, but its progression and spread often correlate with increasing organ involvement and a worse prognosis [4]. The most frequently used method for the assessment of skin involvement, both in clinical studies and in clinical practice, is the modified Rodnan skin score (mRSS). It is based on the physical examination of 17 anatomical regions. The examiner rates the thickening of the skin in each area from 0 to 3 points: no thickening (0 points), slight thickening (1 point), moderate thickening (2 points) or severe thickening (3 points). The scores of the 17 regions are summed to calculate the mRSS, which can range from 0 to a maximum of 51 points [5]. The mRSS has been proven to be well correlated with the histological extent of skin fibrosis and predictive of disease outcomes and survival [5,6,7]. However, despite its simplicity and good validation, there are important drawbacks inherent to the mRSS. The inter- and intra-observer variability of the mRSS is considerably high, ranging between 25 and 54%, and between 12 and 20%, respectively [8]. Furthermore, the sensitivity of the mRSS for detecting small but clinically meaningful changes in skin fibrosis is low [9]. Therefore, for both clinical trials and clinical practice, a more objective, sensitive and reproducible tool to quantify skin involvement in patients with SSc is needed. High-frequency ultrasound (i.e., frequencies ≥ 18 MHz) is an imaging technology that provides an objective method for visualizing the different histological layers of the skin [8]. Its potential in the assessment of skin involvement in SSc is increasingly explored [8,10,11,12].

A recently published systematic literature review by the World Scleroderma Foundation (WSF) Skin Ultrasound Working Group evaluated the available evidence for the three skin ultrasound outcome domains; namely, skin thickness, echogenicity and stiffness. With several clinical studies demonstrating the validity of ultrasound-measured skin thickness against the mRSS and two studies finding positive correlations between ultrasound-measured skin thickness and histological features, skin thickness was evaluated as the ultrasound outcome measure with the most robust supporting evidence [11]. Ultra-high-frequency ultrasound (UHF-US, ≥30 MHz) is a recently introduced diagnostic technique allowing for the high-spatial-resolution imaging of superficial structures. So far, only two studies using UHF-US in the assessment of skin involvement in SSc have been published [13,14].

The aim of this prospective pilot study was firstly to assess and compare skin thickness in the 17 predefined regions of the mRSS using a linear ultra-high-frequency ultrasound probe in patients with SSc and the healthy controls, respectively, and evaluate the interobserver reliability. Secondly, addressing one of the central items of the research agenda for skin ultrasound published by the World Scleroderma Foundation in 2022 [11], we aimed to evaluate the feasibility of skin ultrasound in clinical practice, both with regard to the time needed for image acquisition and analysis, and to the minimum number of skin sites that should be examined.

## 2. Materials and Methods

The study population consisted of 14 in- and outpatients who were prospectively recruited and referred to a tertiary care rheumatology center with known or newly diagnosed SSc between November 2019 and July 2020 (Table 1). The sample size used in the present pilot study for evaluating differences in skin thickness was derived from recommendations in the literature concerning sample size determination for continuous variables in pilot studies [15,16,17].

**Figure 1:**
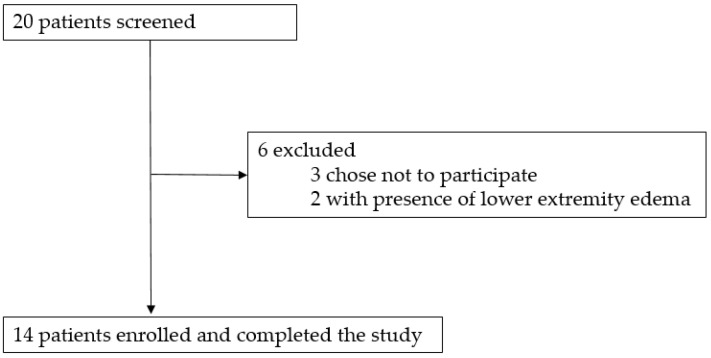
Flow diagram for patient inclusion.

All patients fulfilled the 2013 ACR/EULAR classification criteria [18]. The subset of skin involvement was assessed as diffuse cutaneous SSc (dcSSc) or limited cutaneous SSc (lcSSc) according to the extent of skin involvement [19]. A total of 14 age- and sex-matched healthy individuals were recruited as the control group. Healthy adult subjects were defined as subjects of both genders, >18 years old, without any medical history/records concerning chronic inflammatory/autoimmune rheumatologic diseases and no cardiovascular, liver, kidney, neurological, endocrine or infectious diseases. The exclusion criteria were as follows: topical skin treatment within the last 6 months with drugs (e.g., retinoids, GCs, calcineurin inhibitors, tar) that are suspected or known to influence skin thickness; intensive natural or artificial UV light exposure within the last 4 weeks or during the study period; the presence of lower-extremity edema, which could confound both mRSS and US assessment, and generalized skin diseases or diseases linked to relevant skin involvement (e.g., liver cirrhosis or psoriasis), except for scleroderma (Figure 1). The local ethics committee approved the study (approval code 07-125, approval date 31 August 2011), and written informed consent was obtained from all participants. The study was conducted in compliance with the Declaration of Helsinki, International Conference on Harmonization Good Clinical Practice guidelines and local country regulations.

### 2.1. Clinical Assessment

Demographics and the following clinical and laboratory features of patients and controls were recorded: disease duration, subset of skin involvement (i.e., diffuse cutaneous [dc]/limited cutaneous [lc]), presence of Raynaud’s phenomenon, arthritis, digital ulcers, interstitial lung disease, pulmonary hypertension and other internal organ involvement, treatment, subtypes of antinuclear antibodies (i.e., anti-Scl70, anti-centromere or polymerase antibodies, others) and capillaroscopy. Skin involvement was scored according to the mRSS [5] by an experienced rheumatologist who was not involved in and blinded to the US assessment.

### 2.2. Ultrasound Assessment

Ultrasound image acquisition and measurements of the epidermis, dermis and cutis (epidermis and dermis) in patients and HCs were performed by three rheumatology experts (O.B.K., W.H., B.E.) consecutively and independently with a Canon Aplio i800 system (Canon Medical Systems Corporation, Shimoishigami, Otawara-shi, Tochigi, Japan) for musculoskeletal ultrasound. All sonographers were blinded to the mRSS, which was assessed by an experienced rheumatologist who was not involved in and blinded for the US assessment. An ultra-high-frequency 33 MHz linear probe (iDMS Linear Matrix i33LX9) with the highest possible center frequency was used. The focus was positioned as near as possible to the dermis at 2 mm below the skin surface. The gain was adjusted at 84 dB, and the scans were always performed with the same preset. During the examination, each subject was placed in a supine position with a pillow under their head. All examinations were carried out in a room with an ambient temperature of 20–25 °C. Images were obtained by placing the probe directly over the skin, using a moderate layer of water-based ultrasound gel as a coupling agent between the skin surface and the probe. While the probe was held perpendicular to the skin surface, probe pressure was avoided to ensure that skin thickness did not change due to compression. Measurements were taken at the 17 body sites that represent the anatomical regions of the mRSS:

- Face: longitudinal, 1 cm inferior to the zygomatic arcus on the right cheek.
- Chest: longitudinal, 5 cm caudal to the clavicula on the mid-clavicular line.
- Abdomen: longitudinal, 5 cm above the umbilicus.
- Upper arms (left and right): transverse, at the distance halfway between the epicondylus lateralis and the acromion.
- Forearms (left and right): transverse, dorsal side at the distance two-thirds between the ulnar styloid process and the epicondylus lateralis.
- Hands (left and right): transverse, dorsal side of the third metacarpal bone.
- Fingers (left and right): longitudinal, dorsal side of the basic phalanx of the third finger.
- Thighs (left and right): longitudinal in the middle of the thigh.
- Lower legs (left and right): longitudinal, lateral at distance halfway between the head of the fibula and the lateral malleolus.
- Feet (left and right): longitudinal, over the third metatarsal bone.

**Figure 2:**
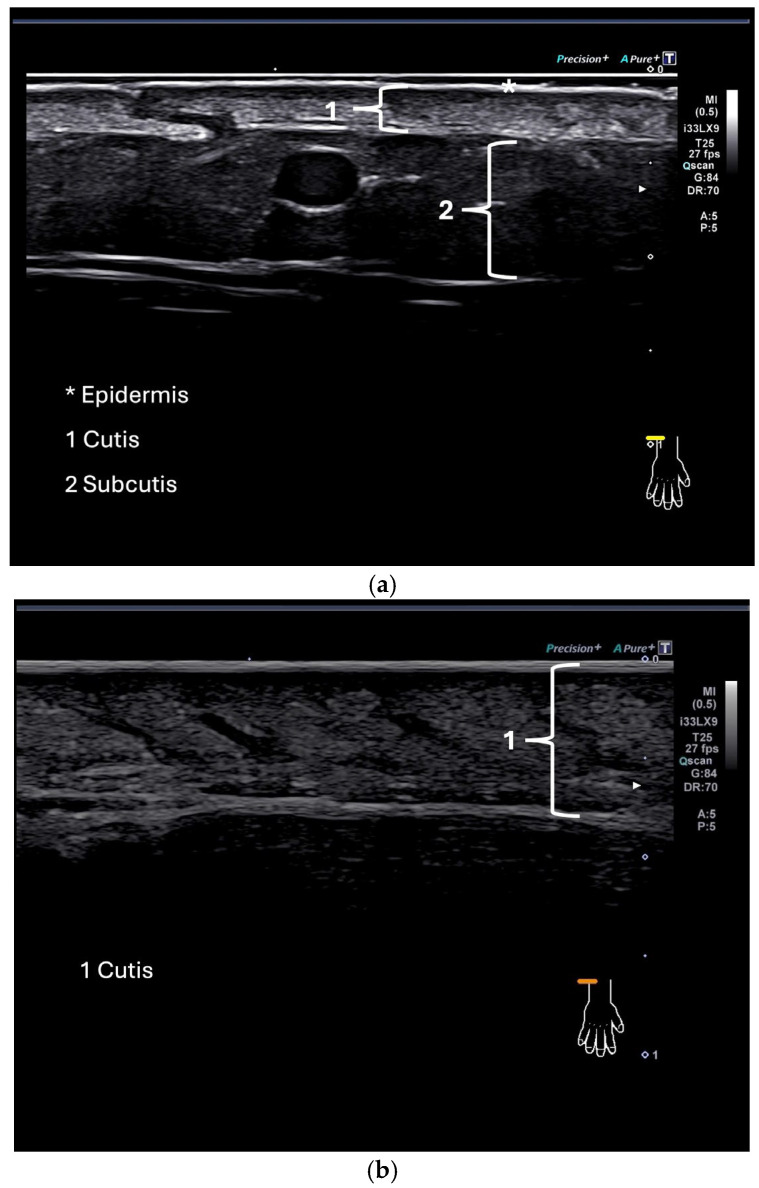
(**a**,**b**) Ultrasound image showing the skin of a healthy control at the dorsum of the forearm (**a**); ultrasound image of the skin of a patient (**b**).

Examples of ultrasound images of the dorsal side of the forearm in a healthy control and a patient with SSc are given in Figure 2.

The caliper was positioned in areas with the clearest detectable discrimination between the dermis and the subdermal layer. The thickness of the epidermis, the dermis and the cutis (epidermis and dermis) was assessed by measuring each three times and calculating the mean using the intima-media thickness measurement tool (IMT) provided by the Canon system. Images and measurements of the dermis, epidermis and cutis from one ultrasound examiner (O.B.K.) were used to compare skin thickness between patients and HCs.

### 2.3. Statistical Analysis

Results were analyzed using the Statistical Package for Social Sciences for Windows, version 28.0 (SPSS, Chicago, IL, USA). A Mann–Whitney U test was used to analyze differences in skin thickness between patients and controls and to analyze differences in skin thickness between patients with clinically assessed absent or mild skin thickening (mRSS 0 or 1) and patients with moderate to severe skin thickening (mRSS 2 or 3). Correlations between UHF-US-assessed dermal thickness and local mRSS were evaluated using Spearman’s rho correlation analysis. Correlation coefficients between 0.0 and 0.3 indicate a weak positive relationship, and values between 0.3 and 0.7 indicate a moderate positive relationship. Values between 0.7 and 1.0 indicate a strong positive linear relationship.

The interobserver reliability for the skin thickness measures obtained from the three sonographers was tested by calculating the intraclass correlation coefficient (ICC; two-way mixed). An ICC value lower than 0.40 was considered poor, 0.40–0.59 moderate, 0.60–0.74 good and 0.75–1 excellent [20]. A *p*-value < 0.05 was considered significant.

## 3. Results

### 3.1. Patient Characteristics

A total of 14 patients suffering from SSc and 14 HCs were enrolled in our study. Twelve (85.7%) patients were female, and two (14.3%) patients were male. The mean (SD, range) age was 68.4 (14.8, 50–81) years in patients with SSc and 67.6 (11.5, 45–86) years in HC. The mean (SD, range) disease duration was 8.6 (5.2, 0–22) years. Most of the patients (*n* = 10, 71.4%) had limited cutaneous disease; only four patients (28.6%) had diffuse cutaneous disease. Ten (71.4%) patients were positive for anti-Scl70 antibodies, and three (21.4%) were positive for anti-centromere antibodies. In one patient, no specific antibodies were detectable. Lung involvement was the most common organ manifestation, with seven (50%) patients suffering from interstitial lung disease and two (14.3%) patients from pulmonary hypertension. One (7.1%) patient had joint involvement, and eight (57.1%) patients had gastrointestinal involvement. A total of 13 out of 14 (92.9%) patients suffered from Raynaud’s phenomenon, and 9 (64.3%) suffered from digital ulcers. Puffy fingers were found in one (7.1%) patient. Capillaroscopy, which was available for all patients, was pathological in all cases. The mean (SD, range) mRSS for patients was 14.9 (13.2, 0–44). The mean mRSS for the controls was 0.0. Ten (71.4%) patients received an immunosuppressant therapy at the time of the ultrasound assessment (one [7.1%] patient received cyclophosphamide as intravenous bolus therapy, three [21.4%] patients received methotrexate and six [42.9%] patients received mycophenolate mofetil). Thirteen (92.9%) patients underwent treatment with a vasodilator, and two (14.3%) patients received low-dose oral glucocorticoids (≤5 mg prednisone per day) (Table 1).

### 3.2. Time Requirement for Ultrasound Assessments

Ultrasound was performed according to the protocol on 14 patients and 14 healthy age- and sex-matched controls in 17 anatomic sites. The mean time spent for ultrasound image acquisition took between 11.8 and 15.4 min (mean time in minutes ± SD: examiner 1: 15.4 ± 5.8, examiner 2: 11.8 ± 2.2; examiner 3: 14.8 ± 4.6). The mean time spent measuring the epidermis, dermis and cutis ranged between 20.1 min and 35.7 min per patient (mean time in minutes ± SD: examiner 1: 34.4 ± 6.9, examiner 2: 20.1 ± 1.5, examiner 3: 35.7 ± 7.1). In comparison, the mean (SD, range) time needed for the assessment of the mRSS by an experienced examiner was 3.4 min (0.8, 2.3–5.4).

### 3.3. Comparison of Skin Thickness Between Patients and Controls

**Figure 3:**
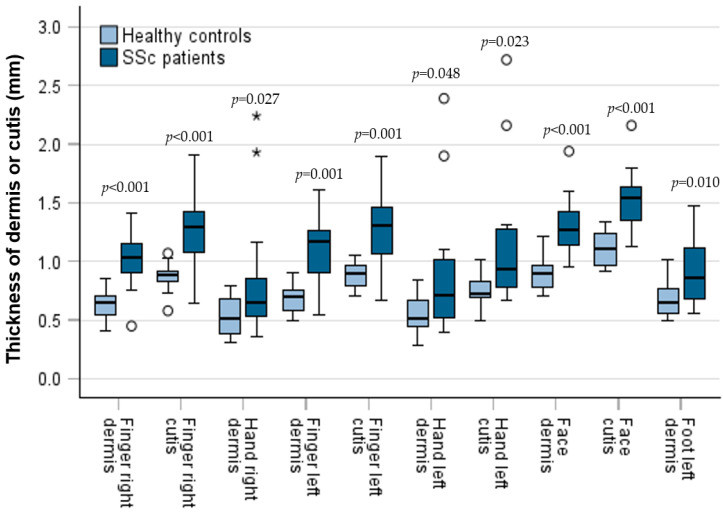
Skin thickness (dermis and cutis) at different anatomical sites of patients and controls as box plots including the 90th, 75th, 50th (median), 25th and 10th percentile. Measurements of patients and controls were compared using the Mann–Whitney test. Points represent outliers; * = represent extreme outliers.

The thickness of the dermis, epidermis and cutis was measured in patients and compared with the measurements of HC. In comparison to HC, dermal thickness was significantly higher in patients at six anatomic sites (right finger: median ± SD: 1.03 ± 0.26 mm; 95% CI: 0.91; 1.15; versus 0.65 ± 0.12 mm; 95% CI: 0.57; 0.71; *p* < 0.001; left finger: median ± SD: 1.17 ± 0.30 mm; 95% CI: 0.91; 1.27; versus 0.70 ± 0.12 mm; 95% CI: 0.59; 0.74; *p* = 0.001; right hand: median ± SD: 0.65 ± 0.56 mm; 95% CI: 0.55; 0.86; versus 0.52 ± 0.16 mm; 95% CI: 0.39; 0.68; *p* = 0.027; left hand: median ± SD: 0.72 ± 0.58 mm; 95% CI: 0.52; 1.01; versus 0.52 ± 0.16 mm; 95% CI: 0.44; 0.67; *p* = 0.048; left foot: median ± SD: 0.86 ± 0.30 mm; 95% CI: 0.67; 1.13; versus 0.67 ± 0.15 mm; 95% CI: 0.55; 0.78; *p* = 0.010; face: median ± SD: 1.28 ± 0.25 mm; 95% CI: 0.67; 1.13; versus 0.90 ± 0.14 mm; 95% CI: 0.78; 0.96; *p* < 0.001). The thickness of the cutis in patients was higher in comparison to healthy controls at the same sites, without reaching significance in the left foot and the right hand (Figure 3). Epidermal thickness did not differ significantly between patients and HCs.

### 3.4. Patients with Diffuse Cutaneous Disease

We analyzed the subgroup of four patients with diffuse cutaneous disease. All the above-mentioned anatomic sites with significant differences in dermal thickness between patients and controls also differed significantly in the subgroup of patients with diffuse cutaneous disease.

### 3.5. Comparison of Clinical (mRSS) and UHF-US Assessment

To investigate whether the sonographic assessment of the dermal thickness of fingers and hands is in line with the clinical evaluation of the skin thickness of the fingers and hands using the mRSS, patients were divided into those with clinically assessed absent to mild skin thickening and those with clinically assessed moderate to severe skin thickening of the fingers and hands. The ultrasound-measured dermal thickness of both the fingers and hands was increased in patients with clinically evaluated moderate to severe (mRSS 2 or 3) skin thickening, reaching statistical significance for both hands and the left finger (Table 2).

At all anatomic sites except for the chest, there were moderate to strong positive correlations between UHF-US-assessed dermal thickness and the local mRSS, with the numerically strongest correlations in the fingers, hands and forearms (Table 3).

### 3.6. Interobserver Reliability

The interobserver reliability for the sonographic measurement of skin thickness was tested by calculating the intraclass correlation coefficient (ICC). With the exception of the face, for all anatomic sites of the mRSS, good to excellent ICC values were obtained (Table 4).

## 4. Discussion

In the last few years, UHF-US has been introduced as a diagnostic technique and a procedural guidance tool allowing for the detailed imaging of skin layers [21] and soft tissue structures due to its unique combination of accessibility, safety and real-time imaging capabilities. It offers high-resolution visualization of superficial structures such as the skin, subcutaneous tissue and fascia. The absence of ionizing radiation and its portability make ultrasound a safe, cost-effective and painless modality that can be used repeatedly and at the point of care [22]. Furthermore, Doppler techniques allow for the assessment of vascular flow, which is essential in evaluating inflammatory activity. Its ability to provide real-time feedback during interventions—such as biopsies, aspirations, or filler injections—significantly improves its procedural accuracy and safety [23].

After a thorough review of the literature, the number of studies using UHF-US in the assessment of skin in SSc is limited. In a pilot cross-sectional study with 21 SSc patients (mean ± SD: mRSS: 5.8 ± 5.2; disease duration: 10.0 ± 8.4 years; 76% of patients with lcSSc, 24% with dcSSc) and 6 HCs, Naredo et al. demonstrated that UHF-US with a frequency range between 50 and 70 MHz, significantly higher than the 33 MHz used in our study, allows for detailed imaging of the skin layers, with good to excellent inter-observer reliability for dermal thickness [13]. The results concerning dermal thickness were heterogenous. Whilst the dermal thickness in the second finger was significantly higher in patients compared to controls, it was significantly lower in the forearm, and there was no significant difference found for the hand. However, the control group was not matched for age and sex. Furthermore, while the percentages of patients with lcSSc and dcSSc were similar, the mean mRSS (mean ± SD: 5.8 ± 5.2) was significantly lower than the mean mRSS in our study (mean ± SD: 14.9 ±13.2). Using UHF-US in 47 SSc patients (mean ± SD: disease duration: 10.8 ± 10.3 years; 58% lcSSc, 23% dcSSc, 19% without scleroderma; mean mRSS not measured) and 15 age- and sex-matched controls, Di Battista et al. found a significantly thicker epidermal layer in patients at all assessed skin sites (dorsal side of the distal, intermediate and proximal phalanx of the second finger; dorsal hand; volar side of the forearm) without significant correlations between UHF-US results and the local or whole-body mRSS [1]. In contrast to our own study, Di Battista and colleagues used a significantly higher ultrasound frequency of 70 MHz. Furthermore, a substantial percentage of patients (19%) were without scleroderma, and patients with digital ulcers were excluded from the study, whereas nine patients in our study suffered from digital ulcers. These differences, indicating the more advanced stage of skin sclerosis of the patients in our study could, at least partially, explain the differing results.

Establishing which skin layer to measure and evaluating the feasibility of skin ultrasound in SSc, with regard to time requirement and the minimum number of sites that should be analyzed, were identified by the World Scleroderma Foundation Ultrasound Working Group as the most important issues to be addressed in future research [11,24]. In the present study, we found a higher skin thickness in SSc patients compared to age- and sex-matched HCs. In the course of our descriptive data analysis, six statistically significant sites, both for the dermis and the cutis (epidermis and dermis), were identified. However, given the exploratory nature of the study, these findings should be interpreted with caution. To account for the issue of multiple-comparisons adjustment, we applied the Holm–Bonferroni correction. After correction, three significant sites remained (right finger, left finger and face; each in both the dermis and cutis). In exploratory studies, it is widely accepted that corrections for multiple testing, such as the Holm–Bonferroni method, are not strictly necessary. These corrections primarily serve to enhance transparency and caution in interpreting statistical significance. Since exploratory analyses focus on hypothesis generation, the results should not be viewed as definitive evidence, but rather as a basis for further confirmatory research. Applying overly strict error rate control in this context may hinder the discovery of meaningful associations.

As epidermal thickness did not significantly differ between SSc patients and HCs and as the interrater reliability for the measurement of the epidermis was poor, we suggest that the dermis should be the skin layer to focus on in ultrasound skin assessment in SSc. Defining the dermis as the target of ultrasound assessment of the skin in SSc would also reflect that the dermis is the main focus of pathological skin changes in SSc [25,26].

The increased dermal thickness in SSc patients, with significant differences in both hands and fingers, reflects the pathophysiology of skin fibrosis in SSc, which typically starts at the distal fingers and toes and progresses proximately [27].

The time needed for the three examiners for ultrasound skin assessment was considerably high, with a mean time for image acquisition ranging from 12 to 15 min and a mean time for the measurement of the epidermis, dermis and cutis ranging from 32 to 51 min. There are only two studies reporting time requirements for skin ultrasound in SSc. Comparable with our findings, for the ultrasound assessment of the 17 mRSS sites restricted to the measurement of the dermal layer, Sulli et al. reported that image acquisition and measurement took 20 to 25 min [28]. For image acquisition of the epidermis and dermis at the 17 mRSS sites, Moore et al. reported a mean time requirement of 20 min [29]. As the reported times for image acquisition and measurements are not practicable in routine clinical settings, we suggest that ultrasound assessment of skin in SSc should be restricted to the dermal layer of the fingers and hands as the focus of pathophysiological changes, and as the site that skin fibrosis typically starts from [25,26,27]. Restricting measurements to 4 instead of 17 skin sites and to 1 instead of 3 skin layers should substantially reduce the time requirement, numerically to one-twelfth of the time needed before the restriction. However, while the restriction of skin ultrasound to the hands and fingers allows for a reliable discrimination of the skin thickening of HCs versus SSc patients because skin thickening of the hands and fingers is a clinical hallmark of SSc, for the recognition of SSc, diagnostic information about the total extent of skin involvement is also important.

In accordance with this proposal, Smith and colleagues recently suggested the restriction of ultrasound assessment in SSc to the measurement of dermal thickness of the left second finger, with a cut-off value discriminating between SSc patients and healthy controls for day-to-day clinical practice [30].

Studies have reported heterogenous results concerning the agreement between local mRSS assessment and objective ultrasound-measured skin thickness [1,18,28,31].

The validity of our data is supported by the significantly increased ultrasound-measured dermal thickness of the fingers and hands in patients with clinically evaluated moderate to severe skin thickening compared to patients with clinically evaluated absent to mild skin thickening. Furthermore, at all anatomic sites except for the chest, there were moderate to strong positive correlations between UHF-US-assessed dermal thickness and local mRSS.

Despite all the mentioned strengths, skin ultrasound also has important limitations. It is operator-dependent, with image quality and interpretation closely tied to the examiner’s experience. However, in our study, a good to excellent inter-observer reliability was shown. This reflects that UHF-US, with its high spatial resolution, allows a very precise and reproducible imaging of skin layers, at least when used by experienced examiners.

Although we have outlined our findings regarding skin ultrasound in SSc, we also want to highlight that ultrasound is an increasingly valuable tool in the assessment of morphea, too. Ultrasound can detect increased dermal thickness and changes in echogenicity. In early disease stages, the loss of normal skin layer architecture, as well as inflammation and early fibrotic changes, can be found. In later stages, ultrasound can help visualize fibrosis and atrophy. Ultrasound is especially useful for monitoring disease progression or therapeutic response, as an alternative to clinical palpation. Moreover, it can assist in identifying subclinical lesions, assessing disease activity and guiding biopsies, as well as determining the disease stage using objective measures [32].

A possible future application of skin ultrasound in patients with SSc could be as follows: the combination of standardized fixtures with technician-controlled robotic ultrasound could offer a reproducible and objective approach to skin imaging. Anatomical supports, such as arm- or headrests, could guarantee consistent patient positioning during examinations. Robotic systems could enable precise and repeatable probe placement with controlled angulation and pressure. Integrating artificial intelligence could further enhance standardization by the real-time segmentation of skin layers and automated thickness measurements. It could facilitate the generation of structured reports, ensuring both efficiency and consistency. This approach could significantly reduce operator dependency and the time required to perform imaging [33,34].

### Study Limitations

Our study has several limitations. First, the relatively small sample size of the cohort limits the generalizability of our results to larger populations of SSc patients. Second, the cross-sectional design of the study does not provide information about the evolution of skin changes with regard to the different stages of skin involvement with an edematous, fibrotic and atrophic phase in temporal sequence. In this regard, further studies should address the UHF-US follow-up of SSc patients to assess longitudinal changes and the responsiveness of UHF-US to skin changes due to pharmacological interventions. Finally, despite the growing interest in the field of rheumatology in skin ultrasound as an imaging tool to quantify skin involvement in patients with SSc, as reflected by the recently published recommendations for the execution and reporting of skin ultrasound from the World Scleroderma Foundation, there remains a lack of standardization and consensus on technical aspects, allowing the comparison of the results from different studies.

## 5. Conclusions

In the present study, UHF-US allowed for the precise, valid and highly reliable measurement of the dermis, with increased dermal thickness in SSc patients compared to age- and sex-matched HC. The increased dermal thickness in SSc patients was confined to 6 out of 17 mRSS sites, with consistently significant differences for both hands and fingers, irrespective of the subgroup of SSc patients (lcSSc or dcSSc). Considering its feasibility in clinical practice, we suggest that US assessment of skin in SSc should be restricted to the dermal layer for detecting pathophysiological skin changes and should focus on the fingers and hands, the sites that skin fibrosis typically starts from.

## Figures and Tables

**Table 1:** Characteristics of patients and HCs. Data are n (%) or mean (SD).

	SSc N = 14	HC N = 14
Age, years	68.4 (14.8)	67.6 (11.5)
Sex		
Female	12 (85.7)	12 (85.7)
Male	2 (14.3)	2 (14.3)
Disease duration, years	8.6 (5.2)	-
Skin involvement		
Diffuse (dc)	4 (28.6)	-
Limited (lc)	10 (71.4)	-
mRSS, points	14.9 (13.2)	0
Antibodies		
Anti-centromere	10 (71.1)	-
Anti-Scl-70	3 (21.4)	-
No specific	1 (2.1)	-
Disease manifestations		
Raynaud’s phenomenon	13 (92.9)	-
Puffy fingers	1 (7.1)	-
Digital ulcers	9 (64.3)	-
Interstitial lung disease	7 (50)	-
Pulmonary hypertension	2 (14.3)	-
Gastrointestinal involvement	8 (57.1)	-
Immunosuppresant therapy	10 (71.4)	-
Mycophenolat mofetil	6 (42.9)	-
Methotrexate	3 (21.4)	-
Cyclophosphamid i.v.	1 (7.4)	-
Low-dose oral GCs	2 (14.3)	-
(≤5 mg prednisone/day)	1 (2.1)	-

Abbreviations: SSc, systemic sclerosis; HC, healthy control; lc, limited cutaneous; dc, diffuse cutaneous; mRSS, modified Rodnan skin score; i.v., intravenous; GCs, glucocorticoids; SD, standard deviation.

**Table 2:** Ultrasound-measured dermal thickness of patients with clinically assessed absent to mild (mRSS 0 or 1) and moderate to severe (mRSS 2 or 3) skin thickening.

	Dermal Thickness, mm	mRSS 0 or 1	mRSS 2 or 3	*p*-Value
Right finger	min	0.45	0.76	
	max	1.15	1.41	
	mean	0.93	1.15	0.200
	SD	0.21	0.24	
Left finger	min	0.54	0.99	
	max	1.27	1.61	
	mean	0.90	1.28	0.025
	SD	0.26	0.19	
Right hand	min	0.36	0.80	
	max	1.17	2.24	
	mean	0.64	1.46	0.032
	SD	0.22	0.64	
Left hand	min	0.40	0.77	
	max	1.10	2.39	
	mean	0.66	1.49	0.023
	SD	0.22	0.68	

Abbreviations: min, minimum; max, maximum; SD, standard deviation; mm, millimeters; mRSS, modified Rodnan skin score.

**Table 3:** Correlation coefficients (Spearman’s rho) of UHF-US-assessed dermal thickness and local mRSS.

Location	Correlation Coefficient	95% CI Lower; Upper	*p*-Value
Right finger	0.76	0.53; 0.89	*p* < 0.001
Left finger	0.85	0.71; 0.91	*p* < 0.001
Right hand	0.64	0.37; 0.83	*p* < 0.001
Left hand	0.61	0.27; 0.83	*p* < 0.001
Upper right arm	0.56		*p* = 0.002
Upper left arm	0.56		*p* = 0.002
Right forearm	0.67	0.39; 0.84	*p* < 0.001
Left forearm	0.61		*p* < 0.001
Face	0.52	0.23; 0.74	*p* = 0.005
Chest	0.02		*p* = 0.902
Abdomen	0.50		*p* = 0.001
Right thigh	0.39		*p* = 0.040
Left thigh	0.42		*p* = 0.025
Lower right leg	0.44	0.11; 0.70	*p* = 0.020
Lower left leg	0.55	0.20; 0.77	*p* = 0.003
Right foot	0.49	0.09; 0.76	*p* = 0.009
Left foot	0.63	0.26; 0.83	*p* < 0.001

**Table 4:** Intraclass correlation coefficient (ICC) values for the anatomic sites of the mRSS.

Location	ICC Dermis	95% CI Lower; Upper	ICC Cutis	95% CI Lower; Upper
Right finger	**0.80**	0.53; 0.93	**0.80**	0.53; 0.93
Left finger	**0.75**	0.42; 0.91	**0.80**	0.51; 0.93
Right hand	**0.84**	0.61; 0.95	**0.87**	0.67; 0.95
Left hand	**0.76**	0.40; 0.92	**0.82**	0.56; 0.94
Upper right arm	**0.91**	0.78; 0.97	**0.87**	0.67; 0.95
Upper left arm	**0.94**	0.85; 0.98	**0.94**	0.85; 0.98
Right forearm	**0.87**	0.68; 0.96	**0.88**	0.70; 0.96
Left forearm	**0.90**	0.75; 0.96	**0.91**	0.78; 0.97
Face	0.56	0.05; 0.83	0.47	0.09; 0.80
Chest	**0.87**	0.68; 0.96	**0.89**	0.74; 0.96
Abdomen	**0.84**	0.60; 0.94	**0.88**	0.70; 0.96
Right thigh	**0.93**	0.83; 0.98	**0.93**	0.82; 0.97
Left thigh	**0.92**	0.80; 0.97	**0.90**	0.76; 0.96
Lower right leg	**0.87**	0.69; 0.96	**0.89**	0.73; 0.96
Lower left leg	**0.92**	0.82; 0.97	**0.92**	0.81; 0.97
Right foot	0.72	0.33; 0.94	0.72	0.35; 0.90
Left foot	**0.89**	0.73; 0.96	**0.85**	0.62; 0.95

## Data Availability

The datasets used and/or analyzed during the current study are available from the corresponding author upon reasonable request.

## Abbreviations

The following abbreviations are used in this manuscript:

UHF-US	Ultra-high-frequency ultrasound
ICC	Intraclass correlation coefficient
SSc	Systemic sclerosis
lcSSc	Limited cutaneous systemic sclerosis
dcSSc	Diffuse cutaneous systemic sclerosis
mRSS	Modified Rodnan skin score
